# Test–retest reliability and predictive utility of a macroscale principal functional connectivity gradient

**DOI:** 10.1002/hbm.26517

**Published:** 2023-10-18

**Authors:** Annchen R. Knodt, Maxwell L. Elliott, Ethan T. Whitman, Alex Winn, Angela Addae, David Ireland, Richie Poulton, Sandhya Ramrakha, Avshalom Caspi, Terrie E. Moffitt, Ahmad R. Hariri

**Affiliations:** ^1^ Department of Psychology and Neuroscience Duke University Durham North Carolina USA; ^2^ Department of Psychology, Center for Brain Science Harvard University Cambridge Massachusetts USA; ^3^ Dunedin Multidisciplinary Health and Development Research Unit, Department of Psychology University of Otago Dunedin New Zealand; ^4^ Department of Psychiatry and Behavioral Sciences Duke University Durham North Carolina USA; ^5^ Institute of Psychiatry, Psychology, and Neuroscience King's College London London UK

**Keywords:** aging, cognitive function, connectivity gradient, cortical hierarchy, functional MRI, test–retest reliability

## Abstract

Mapping individual differences in brain function has been hampered by poor reliability as well as limited interpretability. Leveraging patterns of brain‐wide functional connectivity (FC) offers some promise in this endeavor. In particular, a macroscale principal FC gradient that recapitulates a hierarchical organization spanning molecular, cellular, and circuit level features along a sensory‐to‐association cortical axis has emerged as both a parsimonious and interpretable measure of individual differences in behavior. However, the measurement reliabilities of this FC gradient have not been fully evaluated. Here, we assess the reliabilities of both global and regional principal FC gradient measures using test–retest data from the young adult Human Connectome Project (HCP‐YA) and the Dunedin Study. Analyses revealed that the reliabilities of principal FC gradient measures were (1) consistently higher than those for traditional edge‐wise FC measures, (2) higher for FC measures derived from general FC (GFC) in comparison with resting‐state FC, and (3) higher for longer scan lengths. We additionally examined the relative utility of these principal FC gradient measures in predicting cognition and aging in both datasets as well as the HCP‐aging dataset. These analyses revealed that regional FC gradient measures and global gradient range were significantly associated with aging in all three datasets, and moderately associated with cognition in the HCP‐YA and Dunedin Study datasets, reflecting contractions and expansions of the cortical hierarchy, respectively. Collectively, these results demonstrate that measures of the principal FC gradient, especially derived using GFC, effectively capture a reliable feature of the human brain subject to interpretable and biologically meaningful individual variation, offering some advantages over traditional edge‐wise FC measures in the search for brain–behavior associations.

## Graphical abstract

We demonstrate that a macroscale hierarchical functional connectivity gradient that recapitulates fundamental biological features of the human cortex has higher test–retest reliability than traditional edge‐wise measures, especially when derived from combined resting‐state and task‐based data. Further, gradient measures capture individual differences in aging and cognition.

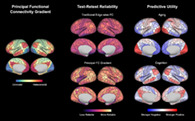

## 1 INTRODUCTION

A primary goal of functional magnetic resonance imaging (fMRI) is to better understand the brain mechanisms that give rise to complex behavior, including the pathophysiology of brain disorders. However, progress toward this goal has been slower than expected, and recent work has drawn attention to challenges arising from underpowered samples and small effect sizes (Marek et al., 2022; Nour et al., 2022) as well as low test–retest reliability of the most commonly studied fMRI measures (Elliott et al., 2020; Noble et al., 2019). In particular, test–retest reliability sets an upper bound on the association that can be observed between two variables (Nunnally, 1959), making it important for researchers examining links between brain and behavior to evaluate the reliability of their measures of interest in order to inform how they design studies and subsequently interpret results. A second key component for positioning fMRI to meaningfully advance the field is the ability to interpret results to the extent that they identify strategies for prevention, diagnosis, or treatment of brain disorders.

Given increasing evidence that complex human behavior depends on distributed networks throughout the brain rather than individual brain regions (Liu et al., 2022; van den Heuvel & Hulshoff Pol, 2010), there is a promising opportunity to search for reliable and behaviorally relevant brain measures that capture putative mechanisms of underlying global functional networks. Studies of intrinsic network structure estimated from resting‐state functional connectivity (rsFC) consistently demonstrate the ability of multivariate global connectivity measures to predict various complex behaviors above chance level, including cognition (Finn et al., 2015; Mansour et al., 2021), personality (Dubois et al., 2018; Hsu et al., 2018; Nostro et al., 2018), and clinical symptoms (Fair et al., 2012; Lake et al., 2019; Wang et al., 2020). Further, recent evidence suggests that estimates of network structure from a combination of resting‐state and task fMRI data, or general FC (GFC), closely mirror those from resting‐state data alone (Cole et al., 2014; Fair et al., 2007; Gratton et al., 2018) while offering the potential for higher reliability and increased predictive utility (Elliott et al., 2019; Greene et al., 2018). However, achieving peak levels of reliability and behavioral prediction accuracy for both rsFC and GFC measures requires a large amount of fMRI data (Elliott et al., 2019; Noble et al., 2019), and acquiring sufficiently long scans can reach prohibitive levels of expense and participant burden. Additionally, there are persistent challenges with interpreting high‐dimensional FC measures (Chen et al., 2022; Tian & Zalesky, 2021), including a long‐standing debate over the meaning of negative correlations induced by data cleaning (Li et al., 2019) and difficulty elucidating how FC emerges from brain structure (Suarez et al., 2020).

In contrast to the bulk of existing research using measures of FC, recent work has shown that the well‐established functional brain networks are not entirely independent but rather are situated along a primary continuous axis of variation that can be revealed by dimension reduction techniques (Margulies et al., 2016). This “principal FC gradient” consists of unitless values at each cortical region that represent the relative position of that region along the axis of variation, where regions that are closer to one another on the gradient have more similar patterns of FC with the rest of the cortex than regions that are farther apart. This gradient closely corresponds with the anatomically established unimodal sensory‐to‐heteromodal association cortical hierarchy (Margulies et al., 2016; Mesulam, 1998). At one end of this hierarchy are unimodal cortices that specialize in performing low‐level sensory and motor processing (e.g., V1, A1, and M1) (Mesulam, 1998). At the opposite end of the hierarchy are heteromodal association cortices, which reach their apex in regions of the default mode networks best known for their contributions to abstract cognitive faculties including episodic memory, semantic cognition, theory of mind, and reasoning about the future (Buckner & DiNicola, 2019; DiNicola et al., 2020). Cortical regions lower in the hierarchy (e.g., V1 and A1) tend to be one or two synapses away from dedicated and direct sensory input (e.g., retinal ganglion cells and cochlear neurons), whereas cortical regions at the apex of the hierarchy (e.g., posterior cingulate and dorsolateral prefrontal cortex), have no direct synaptic connections to sensory neurons and instead are often four or more synapses removed from direct sensory input (Buckner & Margulies, 2019).

In addition, several other known cytoarchitectural and network features differentiate heteromodal association from unimodal sensory cortex in a graded, continuous fashion. For example, as distance from sensory cortex increases, the cortex becomes less myelinated, has more dendritic spines and inhibitory interneurons, maintains information over longer timescales, has more diverse connectivity to the rest of the brain, and has altered patterns of gene expression and receptor distribution (Burt et al., 2018; Demirtas et al., 2019; Goulas et al., 2021; Nakai & Nishimoto, 2020; Schultz et al., 2022; Wang, 2020). These features suggest a hierarchy of sensory abstraction with a greater capacity to flexibly gate an increasing number of inputs, enabled by fewer structural constraints, decreasing responsiveness to external stimulation, and increasing ability to maintain information over time (Buckner & DiNicola, 2019; Fox et al., 2020; Huntenburg et al., 2018; Wang, 2020). Moreover, regions at the apex of this gradient are the most expanded in hominid evolution and take the longest to mature during development (Buckner & Krienen, 2013; Hill et al., 2010; Reardon et al., 2018).

Together, these observations have been combined to form the “tethering hypothesis” of cortical differentiation, which states that the regions of the brain at the apex of the hierarchical sensory‐to‐association gradient allow for uniquely human cognitive abilities because they have become relatively “untethered from sensory signaling hierarchies,” allowing for deeper abstraction and functional flexibility (Buckner & Krienen, 2013). In part, this hypothesis suggests that increasing physical and functional “distance” between sensory and association cortices has allowed for more abstract, uniquely human cognitive abilities to emerge over evolutionary time. Notably, the difference between the regional principal FC gradient values at either end of the axis, or the gradient “range,” represents the functional analog of “distance” between sensory and association areas, where larger values indicate a greater separation between constituent unimodal and transmodal networks and may correspond to a greater degree of “untethering.” Thus, the principal FC gradient appears to represent the functional manifestation of the sensory‐to‐association axis, positioning it as an interpretable proxy measure that allows for the non‐invasive study of the cortical hierarchy in humans. Importantly, the ability to align individual FC gradients to a common template (Coifman & Hirn, 2014; Langs et al., 2015) further allows for direct comparisons between individuals and mapping of individual differences.

Several studies have examined behavioral associations with principal FC gradient measures. Notable findings have included a compressed (or smaller) gradient range in autism (Hong et al., 2019), depression (Xia et al., 2022), and schizophrenia (Dong et al., 2021) as well as during the use of psychedelic drugs (Girn et al., 2022). In contrast, an expanded (or larger) gradient range has been reported in epilepsy (Meng et al., 2021). Others have identified changes in gradient range in response to changing states or task demands (Brown et al., 2022; Cross et al., 2021; Gale et al., 2022; Murphy et al., 2019; Shao et al., 2022; Zhang et al., 2022). Still others have leveraged regional values from one or more FC gradients to predict various behavioral features in multivariate analyses (Bethlehem et al., 2020; Hong et al., 2020; Kong et al., 2022).

While such early studies suggest that the variability of the principal FC gradient is capable of capturing individual differences in both complex normal and abnormal behaviors, proper contextualization of these findings is difficult as there has been a paucity in evaluation of critical psychometric properties of the measure. In particular, prior work on the test–retest reliability of FC gradients is limited to a single study evaluating effects of data processing choices on reliability for the top 100 gradients (Hong et al., 2020). However, this study did not compare gradient reliabilities to that of edge‐wise FC measures, and did not evaluate the reliability of the principal FC gradient range. Here, we assess the test–retest reliability of the principal FC gradient using data collected as part of the publicly available young adult Human Connectome Project (HCP‐YA) and the Dunedin Study. We did so in service of four related goals. First, we tested whether regional principal FC gradient measures are more reliable than traditional edge‐wise FC measures commonly used for studying individual differences. Given that gradient measures are lower dimensional and less prone to the influence of noise, we hypothesized that they would be more reliable. Second, we tested whether regional principal FC gradient measures derived from GFC are more reliable than those derived from rsFC, hypothesizing that as with edge‐wise FC measures (Elliott et al., 2019), gradient measures derived from GFC would be more reliable. Third, we tested whether regional principal FC gradient measures are more reliable for longer scan lengths, again hypothesizing that as with edge‐wise FC measures (Elliott et al., 2019), longer scans would result in higher reliability. Fourth, we evaluated the reliability of the principal FC gradient range for both rsFC and GFC as well as for different scan lengths and hypothesized that results would mirror those with regional gradient measures. Notably, if principal FC gradient measures can achieve good levels of reliability even for shorter scan lengths, that would give them an advantage over other fMRI measures adopted in the search for brain–behavior associations.

After establishing that the reliabilities of principal FC gradient measures indicate their suitability for the study of individual differences, we sought to additionally investigate their predictive utility by testing their relationship with two important measures of health and behavior, namely, aging and cognition. We tested these associations in data from the full HCP‐YA dataset as well as in data collected from 769 members of the Dunedin Study, an ongoing longitudinal investigation of a population‐representative birth cohort. With regard to aging, we used chronological age in HCP‐YA, which ranged from 25 to 35 years old. Given that all Dunedin Study members are the same chronological age (i.e., 45 years at time of scanning), we used a measure of their pace of biological aging instead of chronological age. This measure captures the rate of declining function across multiple organ systems over a 20‐year period and is more closely linked to changes in many behavioral, cognitive, and physical phenotypes than chronological age (Elliott et al., 2021). We additionally sought replication of our findings in a third dataset with a broader range of variability in chronological age, the publicly available HCP‐Aging dataset. In all three studies, cognition was quantified using composite measures of intelligence. Given the “tethering hypothesis” of the emergence of human cognitive abilities via increased distance between sensory and association cortices, we hypothesized that greater cognitive ability would be associated with increases in regional measures of the principal FC gradient in heteromodal cortices, as well as an expanded gradient range. With respect to aging, given that age‐related cognitive decline occurs throughout the lifespan (Salthouse, 2009), that brain aging disproportionately impacts brain areas at the apex of the hierarchical gradient (Douaud et al., 2014), and that a potential corollary of the “tethering hypothesis” is that a reduced gradient range might be a general indicator of poorer brain function, we hypothesized that aging would be associated with reductions in regional values of the principal FC gradient in heteromodal cortices, as well as a compressed gradient range.

## 2 MATERIALS AND METHODS

### 2.1 Datasets

Basic demographics and summary statistics for aging and cognition measures for all datasets are provided in Table 1.

**TABLE 1:** Demographic information and summary statistics for all samples.

Demographics	HCP‐YA test–retest sample (*N* = 32)	Dunedin Study test–retest sample (*N* = 19)	HCP‐YA full sample (*N* = 875^a^)	Dunedin Study full imaging sample (*N* = 769^a^)	HCP‐aging full sample (*N* = 711^a^)
Male sex, % (N)	34.4% (11)	21.1% (4)	46.3% (405)	50.5% (389)	44.3% (315)
Age, M (SD)	30 (3.22)	44.8 (.277)	28.6 (3.73)	45.1 (.681)	60.3 (15.6)
Pace of aging, M (SD)	‐	.899 (.250)	‐	.961 (.269)	‐
Cognition, M (SD)	109.9 (23.3)	98.2 (14.4)	114.5 (19.8)	101.0 (14.6)	109.0 (15.5)
Motion, M (SD)	.157 (040)	.137 (.037)	.156 (.041)	.175 (.058)	.177 (.076)

Abbreviation: HCP‐YA, young adult Human Connectome Project.

^a^ Group *N*s vary slightly for tests of behavioral associations due to missing data.

#### 2.1.1 Human Connectome Project—Young adult

The HCP‐YA is a publicly available dataset that includes 1206 community‐based volunteers with extensive MRI and behavioral measurements (Van Essen et al., 2013). In addition, the entire scan protocol was completed a second time in 45 participants (referred to hereafter as the “test–retest sample”; mean days between scans was approximately 140). All participants were free of current psychiatric or neurologic illness and were 25–35 years of age.

The acquisition parameters and minimal preprocessing of these data have been described extensively elsewhere (Glasser et al., 2013). Briefly, participants underwent extensive MRI measurements that included T1 and T2 weighted structural imaging, diffusion weighted imaging, and nearly 2 h of resting‐state and task fMRI. Then, 1 h of resting‐state fMRI was collected on each participant in four 15‐min scans (1200 time‐points each) split‐up into two scanning sessions over 2 days. In each scan session, the two resting‐state scans were followed by task fMRI (Smith et al., 2013). Across the two sessions, each participant completed seven fMRI tasks described extensively elsewhere (Barch et al., 2013). Briefly, tasks were designed to identify functionally relevant “nodes” in the brain supporting working memory (810 timepoints, 10:02 min); reward processing (506 timepoints, 6:24 min); motor function (568 timepoints, 7:06 min); language (632 timepoints, 7:54 min); social cognition (548 timepoints, 6:54 min); relational processing (464 timepoints, 6:52 min); and emotional processing (352 timepoints, 4:32 min). Altogether, 4800 timepoints totaling 60 min of resting‐state fMRI and 3880 timepoints totaling 48:30 min of task fMRI were collected from each participant.

#### 2.1.2 Dunedin Study imaging sample

The Dunedin Study is a longitudinal investigation of a representative birth cohort (*N* = 1037; 91% of eligible births; 52% male) born between April 1972 and March 1973 in Dunedin, New Zealand (NZ) and eligible based on residence in the province and participation in the first assessment at age 3 years (Poulton et al., 2015). MRI was carried out at age 45 years in 875 Study members, who represented the original cohort on key demographic variables (Figure S1). Additionally, 20 Study members completed the entire scan protocol a second time (mean days between scans = 79).

Each participant was scanned using a Siemens Skyra 3 T scanner equipped with a 64‐channel head/neck coil at the Pacific Radiology imaging center in Dunedin, New Zealand. High resolution structural images were obtained using a T1‐weighted MP‐RAGE sequence with the following parameters: TR = 2400 ms; TE = 1.98 ms; 208 sagittal slices; flip angle = 9°; FOV = 224 mm; matrix = 256 × 256; slice thickness = 0.9 mm with no gap (voxel size 0.9 × 0.875 × 0.875 mm); total scan time = 6:52 min. Functional MRI was collected during resting‐state and four tasks with a series of 72 interleaved axial T2‐weighted functional slices acquired using a threefold multiband accelerated echo planar imaging sequence with the following parameters: TR = 2000 ms, TE = 27 ms, flip angle = 90°, field‐of‐view = 200 mm, voxel size = 2 mm isotropic, slice thickness = 2 mm without gap. 8:16 min (248 timepoints) of resting‐state fMRI were collected immediately before the four task fMRI scans. During the resting‐state scan participants were instructed to stay awake with their eyes open while looking at a gray screen. Participants completed an emotion processing task (200 timepoints, 6:40 min), a color Stroop task (209 timepoints, 6:58 min), a monetary incentive delay task (232 timepoints, 7:44 min) and an episodic memory task (172 timepoints, 5:44 min) for a total of 813 timepoints or 27:06 min of task fMRI. All four tasks are described in detail in the Supplement.

#### 2.1.3 Human connectome project—Aging

The HCP‐Aging dataset is publicly available as part of the ongoing multisite HCP Lifespan study designed to acquire normative neuroimaging and behavioral data for examining changes in brain organization during typical aging and development (Bookheimer et al., 2019). The dataset used in the current study was drawn from the second release (Lifespan HCP Release 2.0), comprised of 725 cognitively healthy older community volunteers (36–100 years old). All participants were screened for a history of neurological, psychiatric, endocrine, genetic, and other serious medical (e.g., diabetes, two or more seizures) disorders, use of psychotropic drugs, and head injuries with loss of consciousness and/or change in mental functioning.

The acquisition parameters and minimal preprocessing of these data have been described extensively elsewhere (Bookheimer et al., 2019; Harms et al., 2018). Briefly, participants underwent extensive MRI measurement that included T1 and T2 weighted structural imaging, diffusion weighted imaging, and close to 40 min of resting‐state and task fMRI. 26 min of resting‐state fMRI were collected on each participant across four 6.5 min runs (488 time‐points each) split‐up into two scanning sessions. In the first scan session, the two resting‐state scans were followed by task fMRI. Each participant completed three fMRI tasks described extensively elsewhere (Bookheimer et al., 2019). Briefly, tasks consisted of visuomotor processing (194 timepoints, 2:46 min), inhibitory control (300 timepoints, 4:11 min), and episodic memory (345 timepoints, 4:47 min). Altogether, 1952 timepoints totaling 26 min of resting‐state fMRI and 839 timepoints totaling 11:44 min of task fMRI were collected from each participant.

### 2.2 Image preprocessing

#### 2.2.1 Human connectome project—Young adult

Preprocessed structural data in MSMSulc surface space (“fMRISurface” pipeline) and functional time series data minimally preprocessed with the “fMRIVolume” pipeline were downloaded from the HCP‐YA database.

Resting‐state fMRI and task fMRI time series images were further processed to limit the influence of motion and other artifacts. Voxel‐wise signal intensities were scaled to yield a time series mean of 100 for each voxel. To remove FC predominantly driven by task‐evoked coactivation, signal due to task structure was added as an additional nuisance covariate to all task fMRI time series and removed using a finite impulse response model (Cole et al., 2019; Fair et al., 2007). Motion regressors were created using each participant's 6 motion correction parameters (3 rotation and 3 translation) and their first derivatives (Jo et al., 2013; Satterthwaite et al., 2013) yielding 12 total motion regressors. Five components from white matter and cerebrospinal fluid were extracted using CompCorr (Behzadi et al., 2007) and also used as nuisance regressors, along with the mean global signal. Images underwent high‐pass filtering with a cutoff of .008 Hz; high frequency signals were retained because removing high frequency signals would have resulted in excessive loss of degrees of freedom due to the very low TR (0.75 s) (Bright et al., 2017; Caballero‐Gaudes & Reynolds, 2017). For censoring high‐motion time points, we followed the empirically derived thresholds of .39 mm frame‐wise displacement or 4.9 units above the median DVARS as recommended (Burgess et al., 2016). Nuisance regression, band‐pass filtering, censoring, and global‐signal regression for each time series were performed in a single processing step using AFNI's 3dTproject. Processed time series images were then converted into CIFTI format and registered to common 32k_fs_LR mesh with MSMSulc (Robinson et al., 2014) using the HCP fMRI Surface Processing Pipeline. Preprocessing was completed for each phase‐encoding direction of each resting‐state and task scan independently, and output was combined to yield time series for computing rsFC and GFC. All final correlation matrices and gradient maps (derivation described in next section) were visually inspected for artifacts.

Six test–retest participants were excluded because they were missing one or more functional scans at either time point, four were excluded because they had less than 40 min of resting‐state data after censoring, one was excluded because they had less than 3.125 min of data on one or more tasks after censoring, and two were excluded because they failed visual inspection of FC correlation matrices, yielding 32 datasets for test–retest reliability analyses. In the full dataset, 244 participants were excluded because they were missing one or more functional scans, 29 were excluded because they had less than 40 min of resting‐state data after censoring, 20 were excluded because they had less than 3.125 min of data on one or more tasks after censoring, and 38 were excluded because they failed visual inspection of FC correlation matrices, yielding 875 imaging datasets for behavior association analyses.

#### 2.2.2 Dunedin Study

Structural MRI data were analyzed using the HCP minimal preprocessing pipeline as extensively detailed elsewhere (Glasser et al., 2013). Briefly, T1‐weighted and FLAIR images were processed through the PreFreeSurfer, FreeSurfer, and PostFreeSurfer pipelines. T1‐weighted and FLAIR images were corrected for readout distortion using the gradient echo field map, coregistered, brain‐extracted, and aligned together in the native T1 space using boundary‐based registration (Greve & Fischl, 2009). Images were then processed with a custom FreeSurfer recon‐all pipeline that is optimized for structural MRI with higher resolution than 1 mm isotropic. Finally, recon‐all output were converted into CIFTI format and registered to common 32k_FS_LR mesh using MSMSulc (Robinson et al., 2014).

Resting‐state fMRI and task fMRI time series images were further processed to limit the influence of motion and other artifacts. Removal of task signal and nuisance regression were conducted as in the HCP‐YA dataset described above. Images were band‐pass filtered to retain frequencies between 0.008 and 0.1 Hz. We investigated a range of frame‐wise displacement cutoffs using QC‐RSFC plots in order to derive the optimal threshold for removing motion artifact as recommended (Power et al., 2014). This investigation led to thresholds of 0.35 mm frame‐wise displacement and 1.55 standardized DVARS. Nuisance regression, band‐pass filtering, censoring, and global‐signal regression for all resting‐state fMRI and task‐fMRI time series were performed in a single processing step using AFNI's 3dTproject. All final correlation matrices and gradient maps were visually inspected for artifacts.

One test–retest participant was excluded due to poor quality data for one of the tasks at the second time point, yielding 19 datasets for test–retest reliability analyses. In the full imaging dataset, 10 participants were excluded because they were missing one or more functional scans, 14 were excluded because of missing anatomical scans or failed QC for the structural processing pipeline, 7 were excluded because they were scanned with a 20‐channel head coil rather than the standard 64‐channel to accommodate larger head circumferences, 61 were excluded because they had too few degrees of freedom remaining after censoring to complete preprocessing, and 14 were excluded because they failed visual inspection of FC correlation matrices, yielding 769 imaging datasets for behavior association analyses, each with at least 23 total min of data remaining after censoring.

#### 2.2.3 Human Connectome Project—Aging

Preprocessed structural data and functional time series data were downloaded from the HCP‐Aging database and processed identically to the HCP‐YA data described above, with the exception of censoring thresholds. As in the Dunedin Study, we investigated a range of frame‐wise displacement cutoffs using QC‐RSFC plots in order to derive the optimal threshold for removing motion artifact as recommended (Power et al., 2014). This investigation led to thresholds of 0.25 mm frame‐wise displacement. At this cutoff, few to no volumes remained as DVARS outliers, so we did not apply an additional DVARS cutoff.

Then, 14 participants were excluded because they were missing one or more functional scans, yielding 711 imaging datasets for all association analyses, each with at least 9.8 total min of data remaining after censoring. Since visual inspection revealed no artifacts and in order to maintain representative variability in age and cognition across this diverse sample, no further exclusions were imposed.

### 2.3 Edge‐wise FC matrices

For all datasets, the 32k_FS_LR space time series data were parcellated into the 360 surface‐based parcels of the HCP Multi‐Modal Parcellation (Glasser et al., 2016). Time series data were subdivided to yield specific data amounts, and resting‐state and task time series were combined for GFC, in each dataset as follows.

#### 2.3.1 Time series for HCP‐YA

Given the large amounts of resting‐state and task fMRI data available in the HCP‐YA dataset, we chose to impose an equal cut‐off of 40 min of post‐censoring data for both rsFC and GFC time series to enable comparisons across modalities for primary analyses. Additionally, to allow investigation of the influence of data amount on reliability, time series data were extracted for each HCP‐YA test–retest participant across a range of amounts (5–40 min of post‐censoring data, in 5‐min intervals). To arrive at a given fixed amount of GFC data, an exactly equal amount of data from all scan types (i.e., 1/8 of total length from each task and resting‐state scan) was combined for total lengths up to 25 min. After this point, equal amounts of each task could no longer be added due to the shorter task scans, so timepoints were selected at random from the pool of remaining timepoints.

#### 2.3.2 Time series for Dunedin Study and HCP‐Aging

In the Dunedin Study and HCP‐Aging datasets, all available post‐censoring time series data were used for both rsFC and GFC in order to maximize available data.

In all datasets, after time series extraction and compilation, pairwise Pearson correlation was applied to create individual edge‐wise FC matrices for each individual and modality.

### 2.4 Gradient generation

All connectivity matrices were then submitted to diffusion map embedding, a nonlinear dimensionality reduction technique (Lafon et al., 2006), to extract subject‐level FC gradients using the Brainspace toolbox (Vos de Wael et al., 2020). The default parameters for the toolbox were used, including retaining only the strongest 10% of FC edges, as in previous studies (Margulies et al., 2016; Paquola et al., 2019; Vos de Wael et al., 2020). This resulted in a principal FC gradient, where a region's position on the gradient reflects a high level of similarity between its FC patterns and those of other regions nearby on the gradient, with decreasing similarity to regions farther away. To enable comparisons across individuals, Procrustes rotations (Coifman & Hirn, 2014; Langs et al., 2015) were used to align individual gradients in all datasets to a group‐level gradient generated using 40 min of resting‐state data from the full HCP‐YA dataset.

To compute the range of the principal FC gradient, gradient values were separately averaged across the 114 unimodal parcels and 246 heteromodal parcels as defined in the Cole‐Anticevic Brain‐wide Network Partition (Ji et al., 2019) and subtracted. Averaging across unimodal and heteromodal regions reduces statistical noise for the range measure and provides a way to ensure that the computation is based on the same regions of the brain for all individuals.

### 2.5 Test–retest reliability

The intraclass correlation coefficient (ICC) was used to quantify the test–retest reliability of both traditional edge‐wise FC and principal FC gradient measures derived from both rsFC and GFC in the HCP‐YA and Dunedin Study test–retest datasets. ICC (3,1) was used in all analyses (Chen et al., 2018). In the HCP‐YA dataset, ICCs were computed for edge‐wise FC matrices and their respective gradients derived from 40 min of rsFC and GFC data as described above. In the Dunedin Study, ICCs were computed for edge‐wise FC matrices and their respective gradients derived from each participant's single resting‐state scan and from the combination of all task and resting‐state scans (i.e., GFC). For traditional edge‐wise FC, ICCs for each parcel were then calculated by averaging the ICCs for all edges from that parcel. In both datasets, t‐tests were used to test for differences between parcel‐wise ICCs for edge‐wise FC and for the principal FC gradient, and between parcel‐wise ICCs for principal FC gradient measures derived from rsFC and from GFC. Parcel‐wise ICCs were additionally calculated across a range of scan lengths (5–40 min) in the HCP‐YA dataset to explore the influence of data amount on reliability as described above. In both datasets, *t* tests were used to test for differences in reliability between unimodal and heteromodal regions (for HCP‐YA, 40 min of data were used). Next, ICCs were calculated for the principal FC gradient range derived from each dataset, modality, and data amount. Finally, given that only the strongest 10% of FC edges were used to generate the principal FC gradient, parcel‐wise ICCs were additionally calculated for FC matrices with only the strongest 10% of edges retained for each node (parcel), and the tests for differences in reliability from the principal FC gradient were repeated.

### 2.6 Associations with age and cognition

To test for the predictive utility of measures of the principal FC gradient, we explored associations between gradient measures and measures of aging and cognition in all three datasets. In HCP‐YA, aging was indicated by chronological age, and cognition was measured using the age‐adjusted NIH toolbox cognitive function composite score, which is derived by averaging Fluid and Crystallized cognition measures and has been shown to have excellent test–retest reliability and strong correlations with established “gold standard” cognitive measures (Akshoomoff et al., 2014; Heaton et al., 2014). Total *N*s for analyses were 875 and 862 for age and cognition, respectively. Given that all Dunedin Study members are the same chronological age, we used a measure of their pace of biological aging instead of chronological age. Pace of biological aging was quantified by tracking declining function in 19 biomarkers indexing the cardiovascular, metabolic, renal, immune, dental, and pulmonary systems across ages 26, 32, 38, and 45 years, referred to as participants' “pace of aging” (Elliott et al., 2021). Cognition was measured with the Wechsler Adult Intelligence Scale–IV at age 45 (Wechsler, 2008). Total *N*s for analyses were 768 and 767 for aging and cognition, respectively. The aging and cognition measures in the HCP‐Aging dataset mirrored those in HCP‐YA (total *N*s were 711 and 597). In each dataset, we used ordinary least squares regressions to predict both regional gradient values and gradient range from aging and cognition in separate models. For brain‐wide associations with regional measures, we corrected for multiple comparisons across the 360 cortical parcels using a false discovery rate (FDR) procedure (Benjamini & Hochberg, 1995). Sex and motion (average frame‐wise displacement) were included as covariates in all analyses. Standardized beta coefficients were generated for reporting, as these provide an interpretable and generalizable measure of effect size, reflecting the number of standard deviations of the predictor variable associated with one standard deviation of change in the outcome variable (Siegel & Wagner, 2022).

The concept and main analyses for this project were preregistered at https://sites.duke.edu/moffittcaspiprojects/files/2021/07/Elliott_2021a.pdf. Code used for performing all statistical analyses is available at https://github.com/HaririLab/Publications/blob/master/Knodt2023HBM_GradientReliability.Rmd. All analyses were checked for accuracy by an independent data analyst who used the manuscript to reproduce and check analyses with an independent copy of the dataset.

## 3 RESULTS

### 3.1 Macroscale principal FC gradient

**FIGURE 1:**
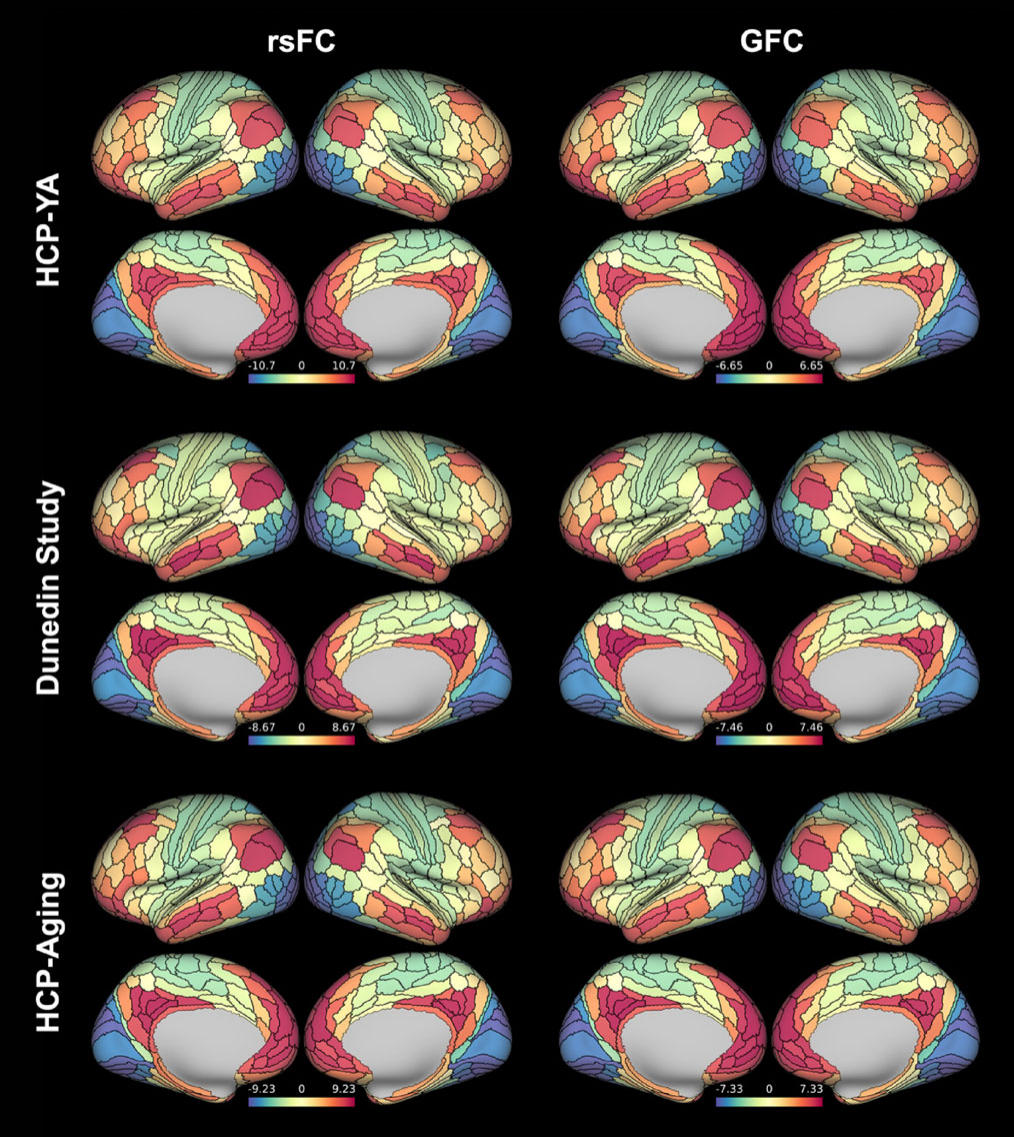
Group‐averaged principal functional connectivity (FC) gradients derived from resting‐state FC (rsFC) and general FC (GFC) for the Human Connectome Project (HCP) multi‐modal parcellation. Gradient values are unitless but allow for relative comparisons.

As expected, the principal FC gradients derived from both rsFC and GFC in the HCP‐YA, Dunedin Study, and HCP‐Aging datasets were anchored in primary sensory regions at one end, transitioning smoothly through unimodal and heteromodal association areas to heteromodal cortex at the apex, replicating previous studies (Bethlehem et al., 2020; Hong et al., 2020; Margulies et al., 2016) (See Figure 1 for group‐averaged maps for rsFC and GFC). Group‐averaged regional (i.e., parcel‐wise) values ranged from −10.71 to 9.65 and − 6.65 to 6.50 for rsFC and GFC, respectively, in HCP‐YA; from −8.67 to 8.44 and −7.00 to 7.46 in the Dunedin Study; and from −9.23 to 8.67 and −7.33 to 7.00 in HCP‐Aging. Note that gradient values are unitless, but since individual gradients for all datasets were aligned to the same template (see Section 2), all values reflect relative positioning along the same axis. In HCP‐YA, the principal FC gradient explained 13.9 ± .7% (mean ± SD) and 17.1 ± 1.3% of individual variance in rsFC and GFC, respectively. In the Dunedin Study, the principal gradient explained 16.5 ± 1.1% and 17.0 ± 1.2% in rsFC and GFC, respectively. In HCP‐Aging, the principal gradient explained 15.3 ± 1.0% and 16.0 ± 1.2%, in rsFC and GFC, respectively. These highly similar patterns across our three datasets are consistent with prior work (Bethlehem et al., 2020; Cross et al., 2021; Hong et al., 2019; Margulies et al., 2016).

### 3.2 Reliability of regional principal FC gradient measures

We used rsFC and GFC data from the HCP‐YA and Dunedin Study test–retest datasets to assess the reliability of regional principal FC gradient measures and test whether it exceeds that of traditional edge‐wise FC.

#### 3.2.1 Reliability of rsFC‐derived principal FC gradient versus edge‐wise FC

**FIGURE 2:**
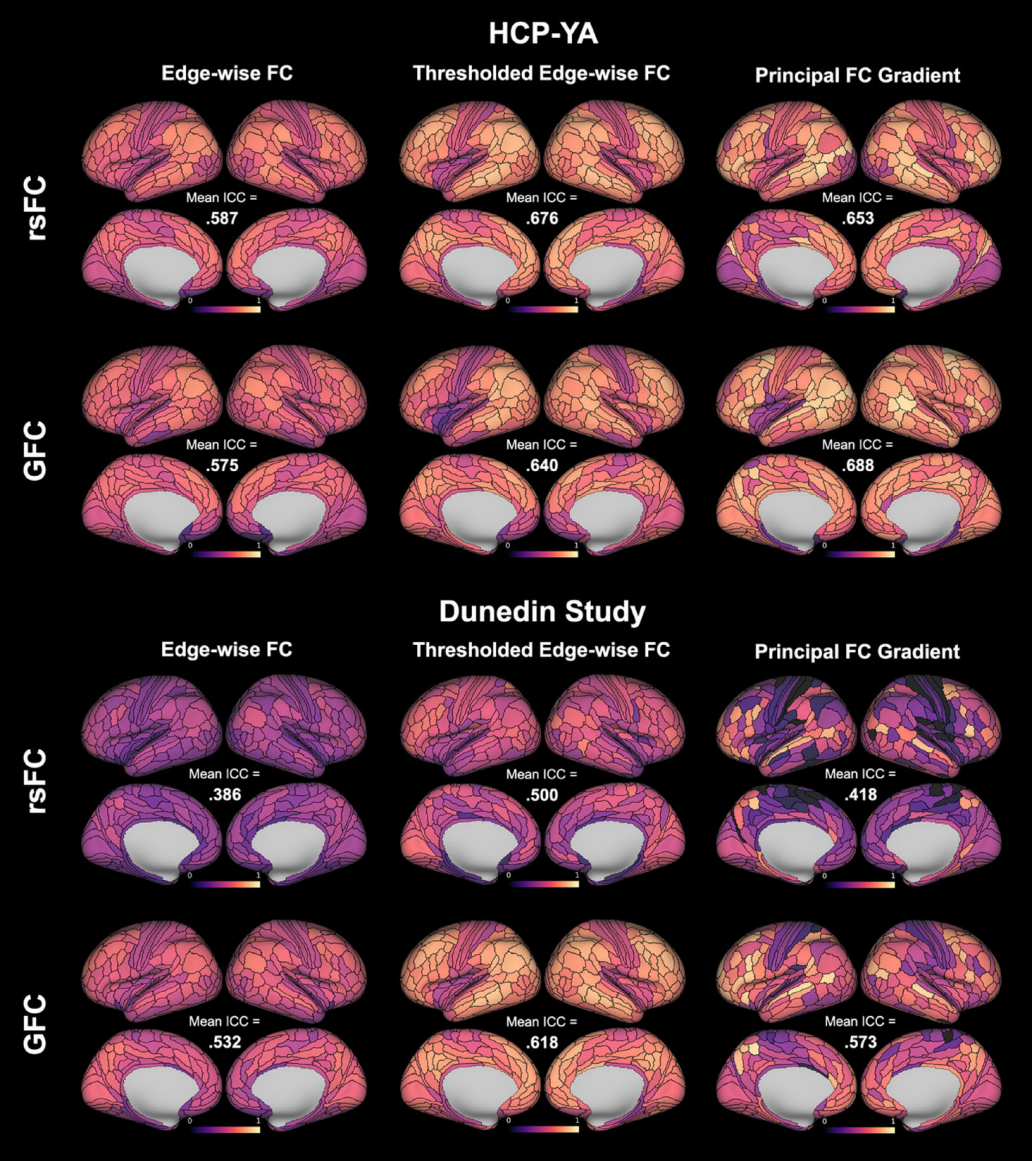
Parcel‐wise reliability of averaged edge‐wise functional connectivity (FC), averaged thresholded edge‐wise FC, and the principal FC gradient derived from resting‐state FC (rsFC) and general FC (GFC) in the young adult Human Connectome Project (HCP‐YA) and Dunedin Study test–retest datasets. For HCP‐YA, 40 min of both rsFC and GFC data were used; for the Dunedin study, ~8 min of rsFC and ~34 min of GFC data were used.

In the HCP‐YA dataset (40 min of data), parcel‐wise ICCs for rsFC‐derived edge‐wise FC (averaged across all participating edges for each parcel) ranged from .316 to .747 (mean across 360 regions = .587; Figure 2). Parcel‐wise ICCs for the principal FC gradient ranged from .175 to .904 (mean across 360 regions = .653). *T*‐tests revealed that parcel‐wise ICCs for the principal FC gradient were significantly higher than for edge‐wise FC (*p* < .001).

In the Dunedin Study dataset (mean data amount = 8.02 min), parcel‐wise ICCs for rsFC‐derived edge‐wise FC (averaged across all participating edges) ranged from .112 to .563 (mean across 360 regions = .386; Figure 2). Parcel‐wise ICCs for the principal FC gradient ranged from 0 to .859 (mean across 360 regions = .418). *T*‐tests revealed that parcel‐wise ICCs for the principal FC gradient were again significantly higher than for edge‐wise FC (*p* = .007).

#### 3.2.2 Reliability of GFC‐derived principal FC gradient versus edge‐wise FC

In the HCP‐YA dataset (40 min of data), parcel‐wise ICCs for GFC‐derived edge‐wise FC (averaged across all participating edges) ranged from .144 to .743 (mean = .575; Figure 2). Parcel‐wise ICCs for the principal FC gradient ranged from 191 to .938 (mean = .688). *T*‐tests revealed that parcel‐wise ICCs for the principal FC gradient were significantly higher than for edge‐wise FC (*p* < .001).

In the Dunedin Study dataset (mean data amount = 33.9 min), parcel‐wise ICCs for GFC‐derived edge‐wise FC (averaged across all participating edges) ranged from .231 to .689 (mean = .532; Figure 2). Parcel‐wise ICCs for the principal FC gradient ranged from 0 to .916 (mean = .573). *T*‐tests again revealed that parcel‐wise ICCs for the principal FC gradient were significantly higher than for edge‐wise FC (*p* < .001).

#### 3.2.3 Reliability of principal FC gradient versus thresholded edge‐wise FC

In the HCP‐YA dataset, parcel‐wise ICCs for thresholded rsFC‐derived edge‐wise FC ranged from .356 to .847 (mean = .676; Figure 2). *T*‐tests revealed that parcel‐wise ICCs for thresholded edge‐wise FC were significantly higher than for the principal FC gradient (*p* = .007). In the Dunedin Study dataset, parcel‐wise ICCs for thresholded rsFC‐derived edge‐wise FC ranged from .109 to .743 (mean = .500; Figure 2). *T*‐tests again revealed that parcel‐wise ICCs for thresholded edge‐wise FC were significantly higher than for the principal FC gradient (*p* < .001).

In the HCP‐YA dataset, parcel‐wise ICCs for thresholded GFC‐derived edge‐wise FC ranged from .176 to .829 (mean = .640; Figure 2). *T*‐tests revealed that parcel‐wise ICCs for thresholded edge‐wise FC were significantly lower than for the principal FC gradient (*p* < .001). In the Dunedin Study dataset, parcel‐wise ICCs for thresholded GFC‐derived edge‐wise FC ranged from .217 to .802 (mean = .618; Figure 2). *T*‐tests again revealed that parcel‐wise ICCs for thresholded edge‐wise FC were significantly higher than for the principal FC gradient (*p* < .001).

#### 3.2.4 Reliability of rsFC‐derived versus GFC‐derived principal FC gradient measures

Next, we tested whether regional gradient ICCs where higher for GFC than for rsFC. *T*‐tests revealed that parcel‐wise ICCs for the principal FC gradient derived from GFC were significantly higher than for rsFC in both the HCP‐YA and Dunedin Study datasets (*p*s < .001).

#### 3.2.5 Effects of data amount on reliability

**FIGURE 3:**
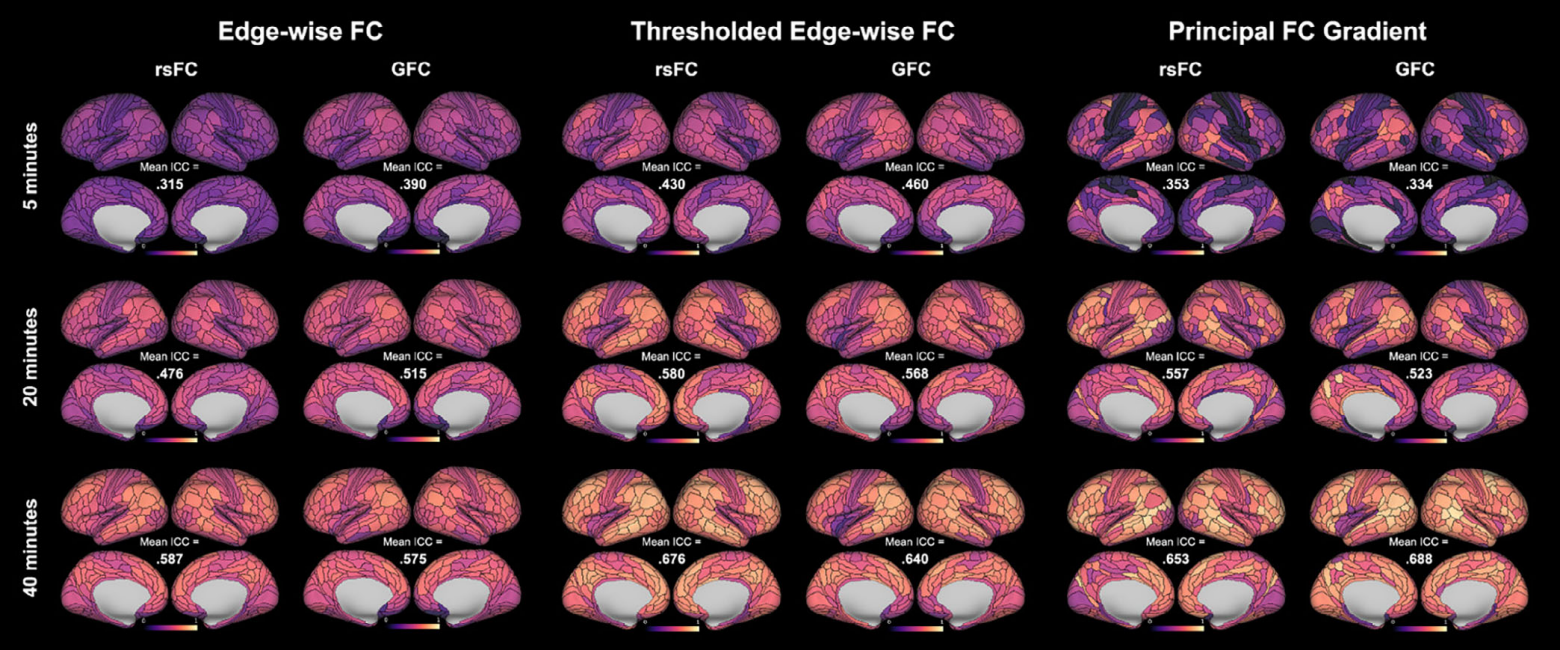
Parcel‐wise reliability of averaged edge‐wise functional connectivity (FC), averaged thresholded edge‐wise FC, and the principal FC gradient derived from resting‐state FC (rsFC) and general FC (GFC) in the young adult Human Connectome Project (HCP‐YA) test–retest dataset, calculated with increasing amounts of data.

**FIGURE 4:**
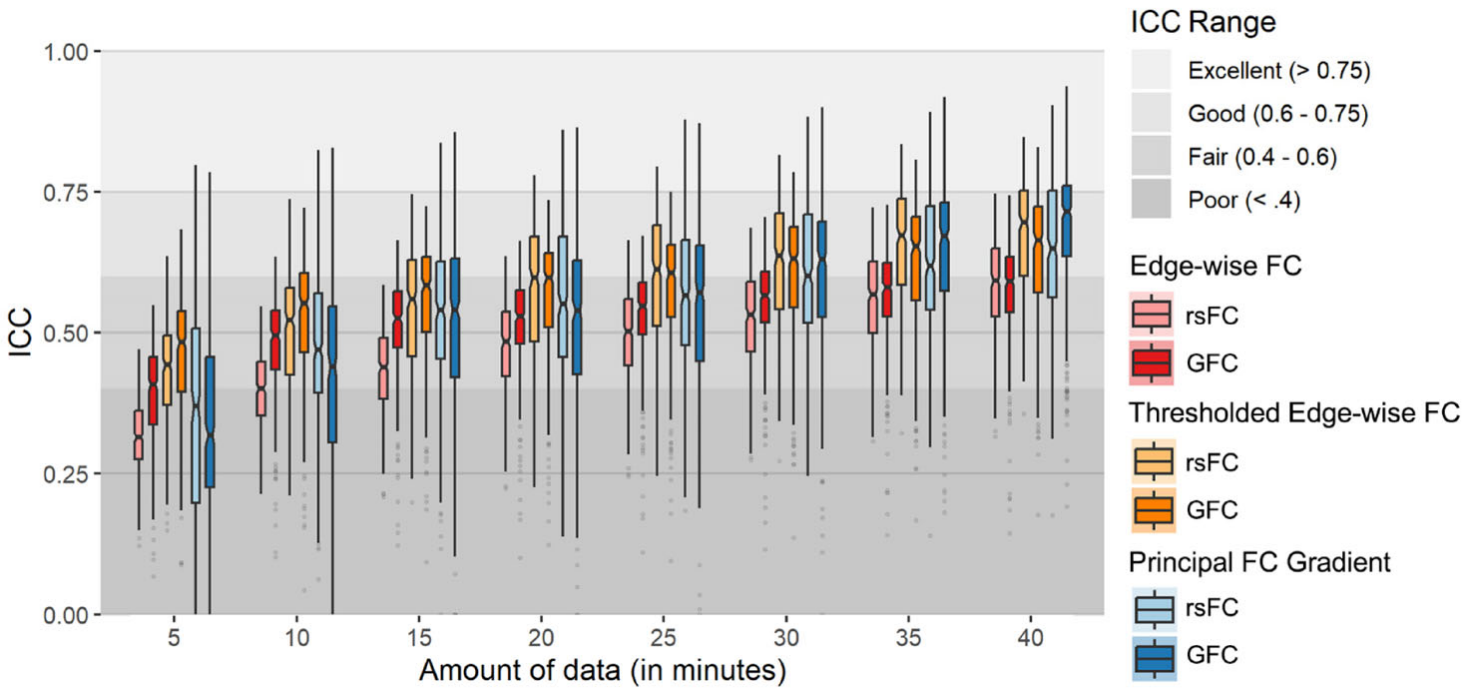
Box plots for the distributions of reliabilities across all functional connectivity (FC) edges (with and without thresholding) and parcel‐wise principal FC gradient values.

Finally, we observed that parcel‐wise ICCs for both edge‐wise FC and the principal FC gradient derived from both rsFC and GFC were higher for larger amounts of data (Figures 3 and 4). This pattern also held for thresholded edge‐wise FC.

#### 3.2.6 Regional differences in reliability

We additionally conducted post hoc tests for differences in ICCs between unimodal and heteromodal regions for both edge‐wise FC values averaged for each parcel and parcel‐wise principal FC gradient values.

In the HCP‐YA dataset, reliability was significantly higher in heteromodal regions for both edge‐wise FC and the principal FC gradient derived from rsFC (*t* = 10.346, *p* < .001 and *t* = 9.631, *p* < .001, respectively). Reliability was significantly higher in heteromodal regions for edge‐wise FC and nonsignificantly higher for the principal FC gradient derived from GFC (*t* = 3.185, *p* = .002 and *t* = 1.434, *p* = .153, respectively).

In the Dunedin Study dataset, reliability was significantly higher in heteromodal regions for both edge‐wise FC and the principal FC gradient derived from rsFC (*t* = 5.040, *p* < .001 and *t* = 4.657, *p* < .001, respectively). Reliability was also higher in heteromodal regions for edge‐wise FC and the principal FC gradient derived from GFC (*t* = 3.608, *p* < .001 and *t* = 7.605, *p* < .001, respectively).

For thresholded edge‐wise FC, in the HCP‐YA dataset, reliability was significantly higher in heteromodal regions when derived from both rsFC and GFC (*t* = 7.875, *p* < .001 and *t* = 2.788 *p* = .006, respectively). In the Dunedin Study dataset, reliability was significantly higher in unimodal regions when derived from rsFC (*t* = −3.071, *p* = .002) and nonsignificantly higher in heteromodal regions when derived from GFC (*t* = .489, *p* = .625).

### 3.3 Reliability of the principal FC gradient range

**FIGURE 5:**
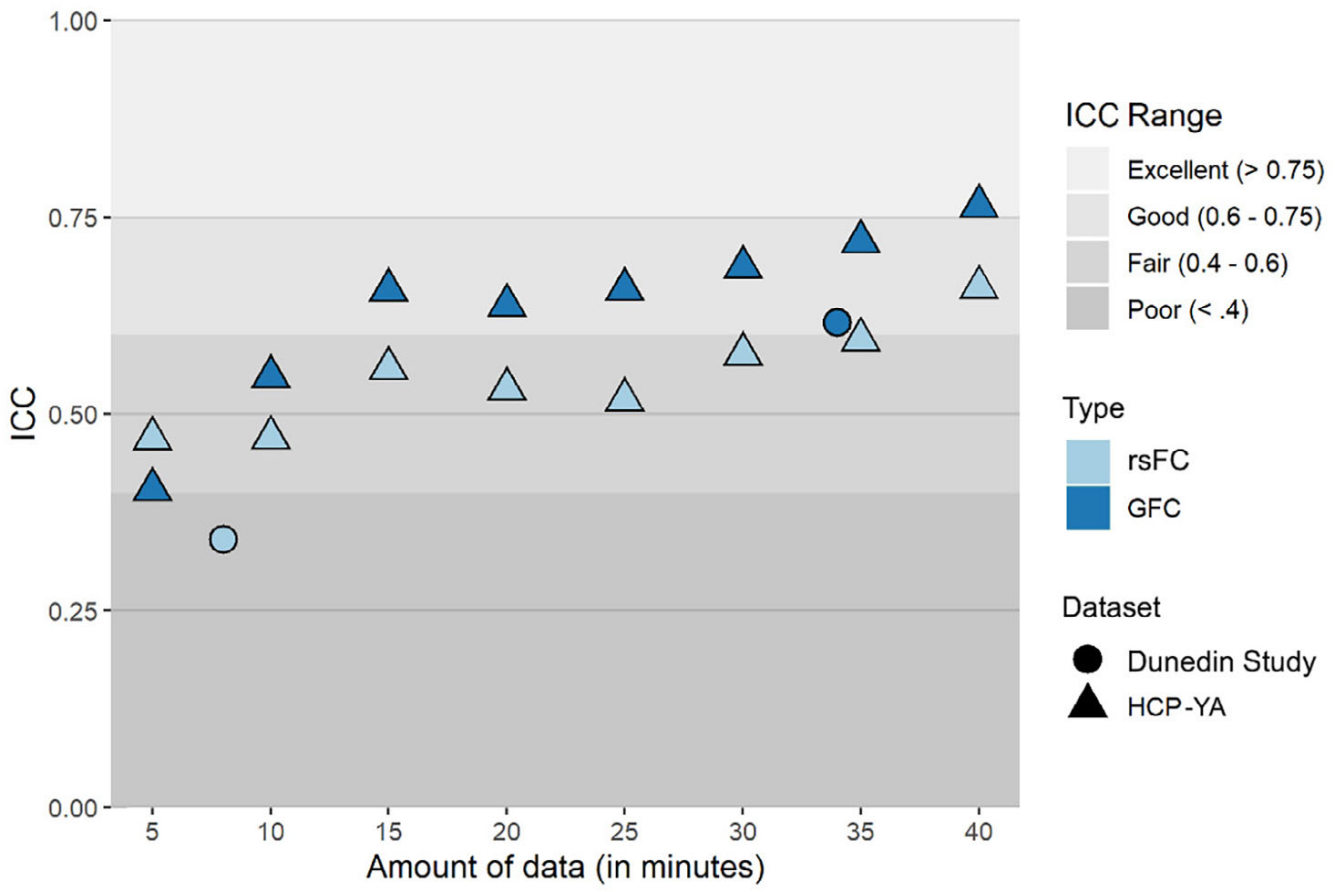
Reliabilities of the principal functional connectivity (FC) gradient range calculated from resting‐state FC (rsFC) and general FC (GFC) data in the young adult Human Connectome Project (HCP‐YA) (triangles) and Dunedin Study (circles) test–retest datasets, plotted by scan length.

Finally, we evaluated the reliability of the principal FC gradient range. In the HCP‐YA dataset, ICCs for the range of the principal FC gradient generally increased with larger amounts of data, reaching .661 for rsFC and .764 for GFC with 40 min of data (Figure 5). In the Dunedin Study dataset, ICCs for the gradient range calculated from an average of 33.9 min of data were .341 for rsFC and .616 for GFC.

### 3.4 Associations with age

After assessing the reliability of principal FC gradient measures, we used the full HCP‐YA and Dunedin Study datasets, as well as the HCP‐Aging dataset, to test their utility in the prediction of two important features of health and behavior, namely aging and cognition.

We first tested whether measures of aging mapped onto regional principal FC gradient values as well as FC gradient range, potentially reflecting the disproportionate effects of aging on heteromodal cortex or mirroring the compression of gradient range observed in mental illness.

#### 3.4.1 Associations with regional principal FC gradient measures

**FIGURE 6:**
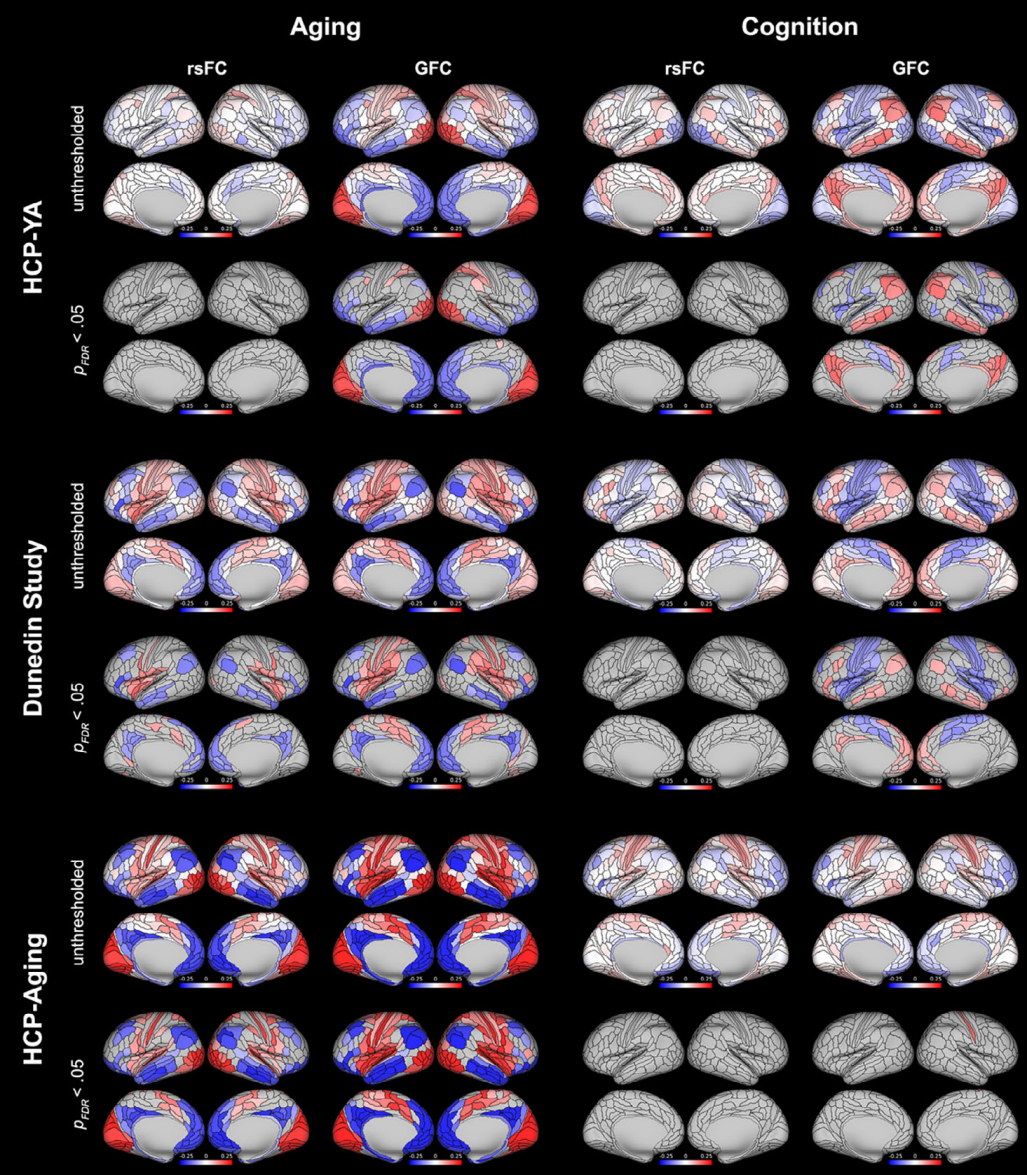
Associations between parcel‐wise principal functional connectivity (FC) gradient values derived from resting‐state FC (rsFC) and general FC (GFC) and measures of aging and cognition in all three datasets. Associations are shown as both whole‐brain unthresholded maps to illustrate the full patterns of associations, as well as thresholded to show only parcels where associations were significant after false discovery rate (FDR)‐correction over 360 tests (*p*_FDR_ < .05).

Parcel‐wise maps of associations between measures of aging and regional values of the principal gradient are shown in Figure 6. Parcel‐wise gradient values derived from rsFC were significantly associated with measures of aging (after FDR correction) in 106 regions (40 of which were positive associations) in the Dunedin Study dataset (*β*s ranged from −.200 to .180), but no regions in the HCP‐YA dataset (*β*s ranged from −.094 to .097). Values derived from GFC were significantly associated with measures of aging in 159 regions (85 positive) in the Dunedin Study dataset (*β*s ranged from −.213 to .173) and 197 regions (82 positive) in the HCP‐YA dataset (*β*s ranged from −.195 to .229). Additionally, gradient values derived from both rsFC and GFC were significantly associated with chronological age in the HCP‐Aging dataset in 261 regions (133 positive) and 301 regions (162 positive), respectively (*β*s ranged from −.243 to .254 and −.348 to .322). Across all datasets, positive associations were concentrated in unimodal regions and negative associations in heteromodal regions, reflecting contractions at the extremes of the cortical hierarchy.

#### Associations with principal FC gradient range

**FIGURE 7:**
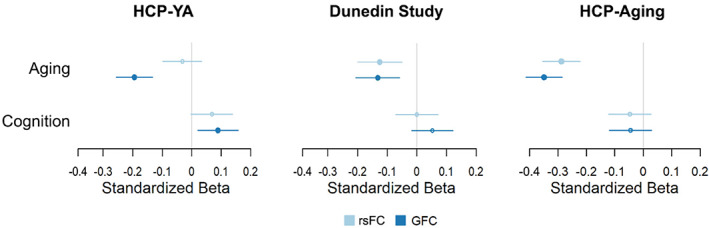
Associations between the principal FC gradient range derived from rsFC and GFC and measures of aging and cognition in all three datasets. Filled circles indicate associations that were significant after FDR‐correction over four tests (*p*_FDR_ < .05). Note that for aging, negative values indicate that older individuals exhibit a more compressed gradient range. For cognition, positive values indicate that individuals with higher cognitive functioning exhibit a more expanded gradient range.

The range of the principal FC gradient derived from rsFC was significantly associated with measures of aging in the Dunedin Study dataset (pace of aging; *β* = −.109, *p* = .004) but not the HCP‐YA dataset (chronological age; though the association was in the same direction: *β* = −.032, *p* = .339; Figure 7). The range derived from GFC was significantly associated with measures of aging in both datasets (*β* = −.120, *p* = .001 in Dunedin and *β* = −.194, *p* < .001 in HCP‐YA). Additionally, the range of the principal gradient derived from both rsFC and GFC was strongly associated with chronological age in the HCP‐Aging dataset (*β* = −.288, *p* < .001 and *β* = −.349, *p* < .001, respectively). Consistent with the cortical distribution of positive and negative parcel‐wise associations, these results reflect an aging‐related contraction in the hierarchy.

### 3.5 Associations with cognition

We next similarly tested whether measures of cognition mapped onto principal FC gradient measures to explore relationships between cognitive ability and regional variation in cortical hierarchy position as well as distance between sensory and association cortices (i.e., FC gradient range, per the “tethering” hypothesis).

#### 3.5.1 Associations with regional principal FC gradient measures

Parcel‐wise gradient values derived from rsFC were not significantly associated with measures of cognition (after FDR correction) in either the HCP‐YA or the Dunedin Study datasets (Figure 6; *β*s ranged from −.119 to .136 and −.116 to .113). Values derived from GFC were significantly associated with measures of cognition in 96 regions (53 positive) in the HCP‐YA dataset (*β*s ranged from −.138 to .169) and 128 regions (49 positive) in the Dunedin Study dataset (*β*s ranged from −.164 to .131). Gradient values derived from GFC were positively associated with age‐adjusted cognition in the HCP‐Aging dataset in 1 region (none for rsFC; *β*s ranged from −.151 to .166 for GFC and −.155 to .139 for rsFC). In the HCP‐YA and Dunedin Study datasets, positive associations were generally concentrated in heteromodal regions and negative associations in unimodal regions, reflecting expansions at the extremes of the cortical hierarchy.

#### 3.5.2 Associations with principal FC gradient range

The range of the principal FC gradient derived from rsFC was not significantly associated with measures of cognition in either the HCP‐YA or Dunedin Study (age‐adjusted cognition composite in HCP‐YA: *β* = .068, *p* = .059; IQ in Dunedin: *β* = 0, *p* = .999; Figure 7). The gradient range derived from GFC was significantly associated with cognition in the HCP‐YA (*β* = .089, *p* = .011) but not the Dunedin Study, though the effect was in the same direction (*β* = .052, *p* = .145). The gradient range was not associated with age‐adjusted cognition in the HCP‐Aging dataset for either measure of connectivity (*β* = −.047, *p* = .218 for rsFC and *β* = −.045, *p* = .238 for GFC). Similarly but to a lesser extent than for associations with aging, these results are consistent with the cortical distribution of positive and negative parcel‐wise associations with cognition, reflecting a cognition‐related expansion in the cortical hierarchy in the HCP‐YA and Dunedin Study datasets.

## 4 DISCUSSION

Here we used parallel analyses in three independent, complementary datasets to first derive the principle FC gradient using both rsFC and GFC, and then evaluate its measurement reliability as well as its predictive utility in explaining individual differences in aging and cognition. In the HCP‐YA and Dunedin Study test–retest datasets, we found that regional reliabilities for the principal FC gradient generally exceeded the commonly accepted “good” threshold of .6 (Cicchetti & Sparrow, 1981) when sufficient data (i.e., at least roughly 30 min) were available. The reliabilities of the gradient measures were consistently higher than those for traditional (i.e., unthresholded) edge‐wise FC measures, were higher for FC derived from GFC as opposed to rsFC, and were higher for longer scan lengths (i.e., larger data amounts). The distance between unimodal and heteromodal cortices, or gradient range, also demonstrated “good” reliability as well as higher reliability when derived from GFC and longer scan lengths. Regional FC gradient values and gradient range were significantly associated with aging in all three datasets (HCP‐YA, Dunedin Study, and HCP‐Aging), and moderately associated with cognition in the HCP‐YA and Dunedin Study, reflecting contractions and expansions of the cortical hierarchy, respectively. Collectively, these results demonstrate that the principal FC gradient effectively captures a reliable feature of the human brain subject to interpretable and biologically meaningful individual variation.

### 4.1 Reliability of the principal FC gradient

Using test–retest data from the HCP‐YA and Dunedin Study datasets, we found good reliability for regional principal FC gradient values. ICCs in the HCP‐YA dataset reached the “good” threshold (i.e., ≥.6) for more than 25% of parcels with as little as 15 min of GFC data (Figures 2 and 4). In contrast, at least 30 min of data were required to reach good reliability for more than 25% of edges with unthresholded edge‐wise FC measures commonly used in the literature. *T*‐tests confirmed that regional principal FC gradient values were consistently more reliable than unthresholded edge‐wise FC values across datasets and modalities. However, when FC edges were thresholded so that only the top 10% of strongest edges were retained (to mirror an intermediate step in the derivation of the principal FC gradient), ICCs reached the “good” threshold for more than 25% of parcels with as little as 10 min of GFC data and were higher than those for regional principal FC gradient (except in the case of GFC in HCP‐YA). These findings are consistent with a previous demonstration of good FC gradient reliability for sufficient amounts of rsFC data (Hong et al., 2020), though to our knowledge, this is the first study to evaluate GFC‐derived gradient reliability and directly compare gradient reliability to edge‐wise FC reliability.

Our observations of greater reliability for the principal FC gradient when compared to unthresholded but not thresholded FC are consistent with prior work and may help to shed additional light on the effects of preprocessing choices on FC reliability. Previous studies have demonstrated increased reliability for edges with the strongest FC (Tozzi et al., 2020), as well as for gradients derived from FC matrices with the highest FC‐strength‐based thresholds (Hong et al., 2020). The present findings of generally lower reliability for the principal FC gradient compared to thresholded FC could be related to previous reports consistently demonstrating that preprocessing pipelines that more effectively reduce artifacts due to motion or non‐brain physiological signals tend to yield less reliable outputs (Birn et al., 2014; Parkes et al., 2018; Shirer et al., 2015). Future work could test whether the application of the diffusion map embedding dimension reduction strategy to the thresholded FC matrix serves to further extract true variance from background noise related to motion or other non‐brain, but reliable, signals. Notably, there remains a lack of consensus on the optimal strategy for striking a balance between reducing artifact while preserving reliability and validity (Ciric et al., 2017; Kassinopoulos & Mitsis, 2022), including considerations related to the implementation of FC‐strength‐based thresholding of FC matrices in studies of individual differences. For example, proportional thresholding (retaining only a given percentage of strongest edges) results in the inclusion of unreliable edges and noisier FC matrices for people with lower average FC, which is often prevalent in patient populations, and absolute thresholding results in different network topologies for different levels of average FC (Ginestet et al., 2011; van den Heuvel et al., 2017; van Wijk et al., 2010; Vasa et al., 2018). Several strategies have been proposed to address these challenges (Ginestet et al., 2011; Vasa et al., 2018), but the lack of a ground truth makes it difficult to identify an optimal approach (Parkes et al., 2018). Importantly, the biological underpinnings of the principal FC gradient may enable its use as a proxy for ground truth in future work seeking to solve this problem. It is worth noting that other preprocessing choices may influence reliability as well. For example, broadening the temporal filter has been associated with increased reliability of edge‐wise FC measures (Shirer et al., 2015), and the application of an alternative dimensionality reduction strategy (i.e., PCA) has been associated with increased reliability for FC gradient measures (Hong et al., 2020). Thus, it is critical that researchers consider the reliability implications of their preprocessing choices when planning and interpreting their analyses.

As expected, we also found that regional principal FC gradient measures derived from GFC were generally more reliable than those derived from rsFC, even for equal amounts of GFC and rsFC data employed in the HCP‐YA dataset. This is consistent with previous demonstrations of increased reliability of GFC for traditional edge‐wise FC measures (Elliott et al., 2019) and suggests that different cognitive states capture complementary trait‐like properties of FC networks that are relevant to the hierarchical organization of the cortex. In the HCP‐YA dataset we observed lower reliability for parcel‐wise principal FC gradient measures derived from GFC than for rsFC with smaller data amounts (<25 min), but this represented a small difference in largely overlapping broad distributions. This potential discrepancy from previous work (Elliott et al., 2019) could be due to slight differences in pre‐processing and brain parcellation. Future work on test–retest datasets with large amounts of resting‐state and task fMRI data is needed to provide additional context for these findings. More consistent with our hypotheses was the observation that, as with edge‐wise FC measures, the reliability of regional principal FC gradient measures was strictly higher for longer scan lengths. This was expected from prior work (Elliott et al., 2019; Hong et al., 2020) and reemphasizes the importance of collecting sufficiently long scans for studies of individual differences. Finally, we found that the reliability of the principal FC gradient range mirrored that of regional gradient values, with higher reliability when derived from GFC and longer scan lengths relative to rsFC and shorter scan lengths. That said, “good” reliability (i.e., ICCs >0.6) was achieved with as little as 15 min of GFC data.

Given that the reliability of a measure sets an upper bound on the size of associations that can be observed with that measure (Nunnally, 1959), this work provides additional support for the use of both regional gradient values and gradient range in studying how individual differences in behavior map onto the functional network structure of the cortex. This is encouraging for fMRI research in light of recent evidence for low test–retest reliability of task activation measures (Elliott et al., 2020) and edge‐wise rsFC measures as revealed by this and previous studies (Noble et al., 2019), especially for low data amounts. The ability of gradient measures to achieve “good” reliability even for smaller data amounts (i.e., 15 min of GFC) suggests they can be leveraged in many existing datasets or collected in future samples where long data collection protocols are not feasible (e.g., children, patients) as a reliable measure of intrinsic network organization for individual differences research. Further, the reliabilities of gradient measures derived from larger amounts of data, especially with GFC, reported here nearly approach those observed for structural MRI measures of the brain including cortical thickness, surface area, and gray matter volume (Elliott et al., 2020). Our additional observation of increased reliability for gradient measures in heteromodal cortices confirms previous findings (Hong et al., 2020) and makes these regions, which support higher‐order processes, especially ripe for the discovery of links between individual differences in brain–behavior associations. Indeed, studies have demonstrated increased individual variability in heteromodal cortices, not only in measures of FC (Benkarim et al., 2021; Mueller et al., 2013) but also functional network topography (Kong et al., 2019) and structure–function coupling (Valk et al., 2022), beginning as early as infancy (Stoecklein et al., 2020).

### 4.2 Gradient associations with age

We found robust associations between the principal FC gradient and aging with all datasets and modalities (with the exception of HCP‐YA rsFC), both at the regional level and with gradient range. Aging‐related reductions in gradient range were driven by movement toward the center of the cortical hierarchy from both ends, that is, increases in gradient values in unimodal regions at the bottom of the hierarchy and decreases in gradient values in heteromodal regions at the top. This was true not only for chronological aging in the HCP‐YA and HCP‐Aging samples, but also for biologically determined pace of aging in the Dunedin Study sample, where participants were all 45 years old at the time of scanning. This suggests that the observed gradient pattern of aging reflects true mechanisms of underlying neurobiological aging, independent of any cohort effects. Notably, effects were stronger for gradient measures derived from GFC data than for those derived from rsFC data, consistent with the higher reliability of those measures. We additionally note that the only analysis for which we did not observe substantial aging‐related effects with gradient measures was that with HCP‐YA rsFC, though the direction of the associations were consistent with the other analyses. This is particularly surprising given the relatively higher reliability of the 40‐min HCP‐YA rsFC data employed here (i.e., higher than the roughly 34‐min Dunedin Study GFC data and considerably higher than the 8‐min Dunedin Study rsFC data, which both demonstrated robust aging effects). We take this observation to further reflect the advantages of GFC for the study of individual differences and the need for further work employing long scan lengths in large datasets.

An aging‐related compression in the cortical hierarchy implies increasing similarity among global connectivity patterns and is consistent with demonstrations of decreased functional network differentiation in brain aging (Chan et al., 2014; Damoiseaux, 2017; Geerligs et al., 2015; Stumme et al., 2020; Wig, 2017), marked by weaker within‐network connectivity and stronger between‐network connectivity (Betzel et al., 2014; Goh, 2011). Nonetheless, our findings represent the strongest evidence to date for aging‐related compression of the principal gradient, given that results from previous studies of FC gradients and aging imply either a lack of a relationship with principal gradient range (Bethlehem et al., 2020) or, in fact, a slight expansion (Setton et al., 2022); however, a third study in a relatively small lifespan sample also found a compression of the gradient in late life, preceded by an expansion into midlife (Nenning et al., 2020). The lack of clear associations in previous studies may be related to smaller samples (Setton et al., 2022) or samples including very young adults and thus less optimized for isolating effects specific to aging (Bethlehem et al., 2020). On the other hand, our consistent findings of aging‐related principal gradient compression in three well‐powered samples, including one focused on later life and one on biological aging, suggest that future efforts to better understand changes in the cortical hierarchy could yield valuable insights into neurobiological aging.

### 4.3 Gradient associations with cognition

Associations between the principal FC gradient and general cognition were somewhat weaker and less consistent than those with aging. Only GFC‐derived gradient range in the HCP‐YA dataset had a significant association with cognition, though the association in the Dunedin Study dataset was in the same direction, and the regional patterns of associations were similar in both the HCP‐YA and Dunedin Study. Given that age has a major influence on cognitive abilities (Salthouse, 2009), adjusting for age may have removed much of the meaningful variance in the HCP‐Aging dataset where there is a much wider age range (36–100 years). In a topographically similar manner to aging (but in the opposite direction), cognition‐related increases in gradient range were driven by movement away from the center of the cortical hierarchy at both ends, that is, decreases in gradient values in unimodal regions at the bottom of the hierarchy and increases in gradient values in heteromodal regions at the top.

While others have demonstrated the ability of FC gradients to predict cognition in multivariate analyses (Hong et al., 2020; Kong et al., 2022), our findings provide a novel mapping between the principal FC gradient and cognition. While previous efforts to map cognition to measures characterizing global properties of FC networks have failed to replicate (Kruschwitz et al., 2018; van den Heuvel et al., 2009), findings with FC gradients usefully inform ongoing research to better understand the brain mechanisms underlying individual variability in this complex behavioral phenotype. An expanded range of the principal FC gradient, indicating increased functional distance between primary sensory and heteromodal association cortices, could, as posited in the “tethering hypothesis” (Buckner & Krienen, 2013), enable greater functional flexibility of association cortices and a wider span of possible network configurations facilitating more flexible cognition. This would seem consistent with evidence for a relationship between network segregation and cognitive ability (Chan et al., 2014; Wig, 2017). However, this is potentially inconsistent with a recent study performing a hierarchical analysis in the HCP‐YA dataset reporting that general cognitive ability maps more readily onto global integration (Wang et al., 2021). Nonetheless, the principal FC gradient offers a parsimonious and interpretable perspective for future efforts to better understand the brain mechanisms of cognition.

### 4.4 Limitations

Our findings should be interpreted in light of several limitations. First, we conducted all analyses using time series data that had been parcellated using a common group‐level parcellation scheme, which has been shown to mask important individual differences due to variation in functional topography (Laumann et al., 2015; Wang et al., 2015). As such, our results likely represent a lower bound on the ability of the principal FC gradient to predict individual differences in aging and cognition. Second, other methods of data acquisition (e.g., multi echo) and preprocessing strategies (as described earlier), as well as multivariate methods, have been shown to increase reliability of fMRI measures, and we refer the interested reader to previous work for thorough evaluations of the effects of these factors on reliability (Hong et al., 2020; Lynch et al., 2020; Yoo et al., 2019). However, our focus on acquisition, processing, and analysis strategies currently typical for the field provides better context for existing and ongoing studies of individual differences in the principal FC gradient and edge‐wise FC. Third, it has been shown that secondary FC gradients are also reproducible across datasets and relevant to behavior (Bethlehem et al., 2020; Brown et al., 2022; Girn et al., 2022; Setton et al., 2022; Sydnor et al., 2021), and very recent work has demonstrated that 40 or 50 gradients are optimal for predicting behavior (Kong et al., 2022). We chose to focus on better understanding the behavioral relevance of the principal FC gradient because of its clear biological underpinnings and consistently maximal explanation of variance in target constructs (Huntenburg et al., 2018). Future work should further explore these questions with additional gradients.

## 5 CONCLUSION

We have shown that the principal FC gradient, especially derived using GFC, is more reliable than traditional edge‐wise FC measures and captures important individual differences in associations between the brain and measures of health and behavior. The ability of this noninvasively measurable proxy of the sensory‐to‐association axis of the human cortex to capture meaningful individual differences offers promise for further insight into how the hierarchical organization of the brain gives rise to complex human phenomena including aging and cognition.

## FUNDING INFORMATION

This research received support from US‐National Institute on Aging grants R01AG069939, R01AG032282, and R01AG049789 and UK Medical Research Council grant MR/P005918/1. The Dunedin Multidisciplinary Health and Development Research Unit is supported by the New Zealand Health Research Council (Programme Grant 16‐604) and New Zealand Ministry of Business, Innovation and Employment (MBIE).

## CONFLICT OF INTEREST STATEMENT

The authors report no biomedical financial interests or potential conflicts of interest.

## Supporting information


**DATA S1:** Supporting Information.

## Data Availability

Dunedin Study data are available via managed access (https://moffittcaspi.trinity.duke.edu/research).
