# Supplementary material for: Test–retest reliability and predictive utility of a macroscale principal functional connectivity gradient
Source: Hum Brain Mapp. 2023 Oct 18;44(18):6399–417. doi: 10.1002/hbm.26517 (PMC10681655; doi:10.1002/hbm.26517)
Supplement: Supplementary file 1 — DATA S1: Supporting Information. [file HBM-44-6399-s001.docx]

# Supplementary Information for

**Test-retest reliability and predictive utility of a principal functional connectivity gradient in capturing individual differences in brain-behavior associations**

Knodt AR, Elliott ML, Whitman E, Winn A, Addae A, Ireland D, Poulton R, Ramrakha S, Caspi A, Moffitt TE, Hariri AR

**Supplemental Experimental Procedures**

**Task fMRI paradigms**

*Human Connectome Project.* Task fMRI paradigms for the Human Connectome Project are described extensively in (Barch et al., 2013).

*Dunedin Longitudinal Study*

*Emotion processing task.* The task consists of four blocks of a perceptual face-matching task interleaved with five blocks of a sensorimotor control task. The Dunedin Study version of this task consists of one block each of fearful, angry, surprised, and neutral facial expressions presented in a pseudorandom order across participants. During face-matching blocks, participants view a trio of faces and select one of two faces (on the bottom) identical to a target face (on the top). Each face processing block consists of six images, balanced for gender, all of which were derived from a standard set of pictures of facial affect. During the sensorimotor control blocks, participants view a trio of simple geometric shapes (circles and vertical and horizontal ellipses) and select one of two shapes (bottom) that are identical to a target shape (top). Each sensorimotor control block consists of six different shape trios. All blocks are preceded by a brief instruction ("Match Faces" or "Match Shapes") that lasts 2 s. In the task blocks, each of the six face trios is presented for 4 s with a variable interstimulus interval (ISI) of 2-6 s (mean = 4 s) for a total block length of 48 s. A variable ISI is used to minimize expectancy effects and resulting habituation and maximize amygdala reactivity throughout the paradigm. In the control blocks, each of the six shape trios is presented for 4 s with a fixed ISI of 2 s for a total block length of 36 s.

*Stroop task.* In this version of the Stroop task, participants identify the color of a target word in the center of a screen by selecting 1 of 4 identifier words. Selections are made by pressing 1 of 4 buttons on a button box, with each button matching an identifier word on the screen (e.g., index finger button 1 = identifier word on the far left, etc.). In congruent trials, targets were in colors congruent with the target words; in incongruent trials, targets are in colors incongruent with the targets. The four identifier words are in white in both conditions. After a 2 s fixation lead in, participants completed three 60 s blocks of congruent trials interleaved with three 60 s blocks of incongruent trials. Both conditions are followed by an 8 s fixation period for a total task time of 6 min and 50 s. Each block contains 12 trials, each consisting of 2 s stimuli, 1 s feedback, and a variable inter stimulus interval averaging 2 s.

*Monetary incentive delay (MID) task.* This version of an event-related MID task consists of 12 eight second trials for each of 3 conditions, presented in a pseudo-random order, for a total of 36 trials. Conditions consist of potential $1 reward, potential $5 reward, and no monetary outcome. On reward trials, participants could win money by pressing a button during the presentation of a target. During each trial, participants see either a green dollar amount cue (reward conditions) or a white “$0” (neutral condition) (cue, 2 seconds), then fixate on an “x” as they wait for a variable interval (delay, 2250–3000 ms), and then respond with a button press to a white target triangle that appears for a variable length of time (target, 70-680 ms) with a button press. Feedback (feedback, 2 seconds), which follows the disappearance of the target, notifies participants of whether they had successfully responded to the target with either the word “HIT” or “MISS”, and indicates their cumulative total at that point. Initial task difficulty (i.e. duration of the target) is based on reaction times collected during a practice session before scanning, and an adaptive algorithm was employed to adjust the target’s duration such that each participant should succeed on ~66% of his or her target responses. Trials are separated by a variable (2-6 seconds) inter-trial interval. fMRI volume acquisitions are time-locked to the offset of each cue and thus were acquired during anticipatory delay periods.

*Memory encoding task.* This task consists of the encoding and subsequent recall of novel face-name pairs. A distractor task (odd/even number identification) is interleaved between encoding and recall blocks to prevent maintenance of information in working memory. During each of four encoding blocks, subjects view six novel face-name pairs for 3.5 seconds each. During each of four recall blocks, subjects view six faces each presented for 2 seconds and immediately followed by an incomplete name fragment for 1.5 seconds during which they are required by forced-choice to determine if the fragment is correct or incorrect. A 1.5 second inter-trial interval is used during recall blocks. During each of four distractor blocks, subjects view six different numbers for 3.5 seconds each and are required to determine if the numbers are odd or even.

**Supplemental Figures**


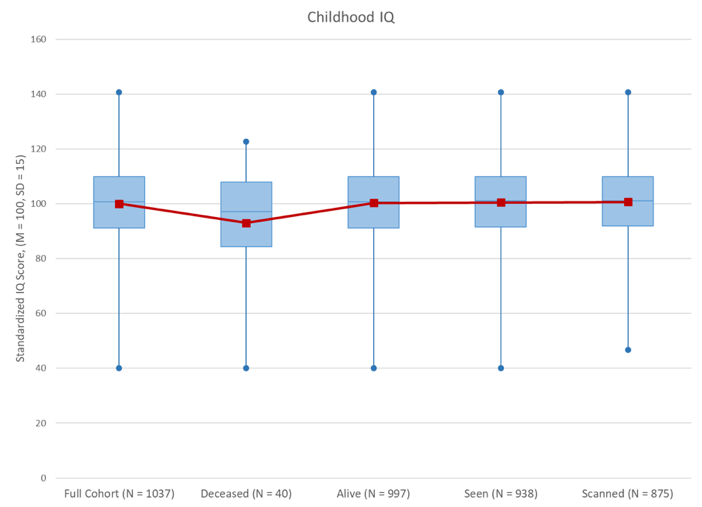

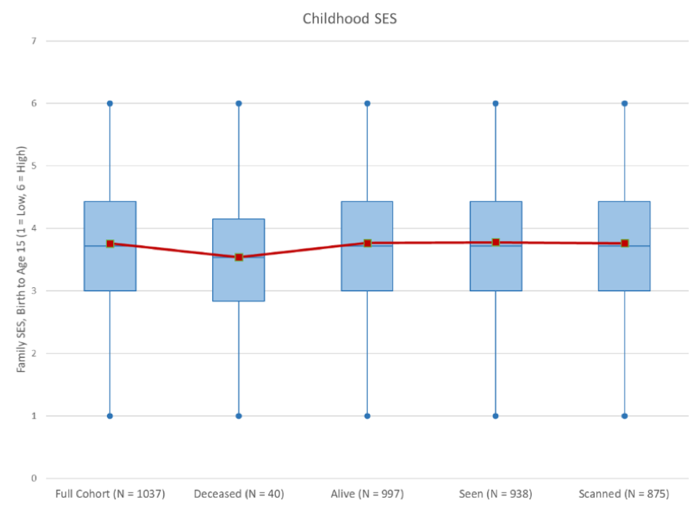


**Figure S1**. Attrition analysis using (A) childhood intelligence quotient (IQ) and (B) socioeconomic status (SES) to determine whether participants in the Phase 45 data collection were representative of the original cohort. No significant differences in childhood IQ were found between the full cohort, those still alive, those seen at Phase 45 or those scanned at Phase 45. Those who were deceased by the Phase 45 data collection had significantly lower childhood IQ’s than those who were still alive (t = 2.09, p = 0.04). No significant differences were found between the full cohort, those deceased, those alive, those seen at Phase 45 or those scanned at Phase 45 on childhood SES.
